# Ventricular and Choroid Plexus Morphological Alterations and Their Associations With Peripheral Biomarkers in Behavioral Variant Frontotemporal Dementia

**DOI:** 10.1002/brb3.71892

**Published:** 2026-11-05

**Authors:** Ailing Yue, Qianqian He, Hua Lu, Yufei Chen, Jiahui Hou, Yuxuan Xu, Liyong Wu, Min Chu

**Affiliations:** ^1^ Department of Neurology Xuanwu Hospital Capital Medical University Beijing People's Republic of China; ^2^ National Clinical Research Center for Geriatric Diseases Beijing People's Republic of China

**Keywords:** choroid plexus, frontotemporal dementia, ventricular system

## Abstract

**Background:**

Frontotemporal dementia (FTD) is a neurodegenerative disorder and a major cause of early‐onset dementia. Alterations in ventricular and choroid plexus volumes have been reported in several neurodegenerative diseases. The morphological alterations of the ventricular system and choroid plexus in behavioral variant frontotemporal dementia (bvFTD) and their associations with clinical and pathophysiological features remain insufficiently explored.

**Methods:**

Ventricular volumes, ventricular asymmetry index, and choroid plexus volume were evaluated in 120 bvFTD patients and 87 normal controls (NC) using T1‐weighted MRI. Correlations with clinical scales, brain volumes, and plasma biomarkers were analyzed, followed by mediation analysis and assessment of diagnostic efficacy.

**Results:**

The bvFTD patients exhibited significant ventricular enlargement (most pronounced in the inferior lateral ventricles) and choroid plexus hypertrophy. Ventricular volumes were correlated with gray matter atrophy, behavioral impairment scores, and plasma biomarkers (e.g., glial fibrillary acidic protein, GFAP). Mediation analysis showed that GFAP mediated the association between ventricular enlargement and behavioral abnormalities. The inferior lateral ventricle volume showed the highest diagnostic efficacy (AUC = 0.9550).

**Conclusion:**

Significant ventricular enlargement and choroid plexus hypertrophy are involved in bvFTD. The inferior lateral ventricle serves as a highly sensitive diagnostic indicator, and GFAP‐mediated inflammation plays a pivotal role in linking ventricular morphological changes to behavioral deficits.

## Graphical abstract

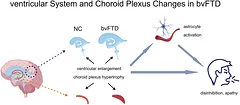

**Abbreviations**
ADL: activities of daily living
bvFTD: behavioral variant frontotemporal dementia
CDR: clinical dementia rating
CHI3L1: chitinase‐3‐like protein 1
CHIT1: chitotriosidase‐1
CSF: cerebrospinal fluid
CX3CL1: fractalkine
FBI: Frontal Behavior Inventory
FTD: frontotemporal dementia
FTLD: frontotemporal lobar degeneration
GFAP: glial fibrillary acidic protein
MMP‐9: matrix metalloproteinase‐9
MMSE: Mini‐Mental State Examination
MoCA: Montreal Cognitive Assessment
NACC: National Alzheimer's Coordinating Center
NC: normal controls
NFL: neurofilament light
NPI‐Q: Neuropsychiatric Inventory Questionnaire
PDGF‐BB: platelet‐derived growth factor‐BB
TGF‐β1: transforming growth factor‐beta 1
t‐tau: total Tau
VCAM‐1: vascular cell adhesion molecule‐1
VEGFA: vascular endothelial growth factor A

## 1 Introduction

Frontotemporal dementia (FTD) is an early‐onset dementia characterized by progressive neurodegeneration predominantly localized to the frontal and temporal lobes. The behavioral variant of FTD (bvFTD) represents the most prevalent subtype, clinically distinguished by significant behavioral abnormalities and frontotemporal atrophy (Rascovsky et al. 2011; Bang et al. 2015). Most of the previous research has focused on alterations of cortical regions; however, the potential contribution of the ventricular system to the pathogenesis of FTD remains underexplored.

The structural architecture of the ventricular system encompasses ventricular volume and asymmetry, and the volume of the choroid plexus (Ge et al. 2024; Lin et al. 2025). Accumulating evidence associates ventricular system abnormalities, such as ventricular size and choroid plexus volume, with neurodegenerative diseases, including Alzheimer's disease (AD), FTD, dementia with Lewy bodies (DLB), and Parkinson's disease with mild cognitive impairment (PD‐MCI) (Lin et al. 2025; Hong et al. 2025; Xia et al. 2025; Tavares et al. 2019; Dalaker et al. 2011; Planas‐Ballvé et al. 2024; Sapkota et al. 2018; DeCarli et al. 1992; Jeong et al. 2005). Notably, alterations in ventricular volume have been observed in the prodromal phase of FTD, highlighting the critical importance of elucidating the roles of the ventricular system in FTD (Tavares et al. 2019; Fyfe 2019). Furthermore, the choroid plexus has recently emerged as a significant marker reflecting CSF production and glymphatic system function; in both AD and FTD, it has been confirmed to hypertrophy and correlate with clinical features (Lin et al. 2025; Xia et al. 2025; Jiang et al. 2023).

Choroid plexus enlargement has been associated with white matter microstructural damage and systemic inflammation in AD, suggesting a potential link between ventricular system alterations and neuroinflammatory and neurodegenerative processes (Xia et al. 2025). Increasing evidence suggests that neuroinflammation, vascular dysfunction, and neuronal injury are involved in FTD (Swift et al. 2021). Previous studies have reported elevated levels of biomarkers associated with astrocytic and microglial activation, including glial fibrillary acidic protein (GFAP), chitinase‐3‐like protein 1 (CHI3L1), and galectin‐3, in patients with FTD (Benussi et al. 2020; Villar‐Piqué et al. 2019; Erichsen et al. 2025). Alterations in vascular‐related biomarkers, such as vascular cell adhesion molecule‐1 (VCAM1), matrix metalloproteinase‐9 (MMP‐9), and vascular endothelial growth factor A (VEGFA), have also been reported (Simrén et al. 2026; Lorenzl et al. 2008; Janelidze et al. 2017; Tuna et al. 2018). Furthermore, neurofilament light (NFL), a marker of axonal injury, is markedly elevated in FTD (Liampas et al. 2024). Some of these biomarkers have also been associated with clinical manifestations, glymphatic function, and structural brain alterations, suggesting potential relationships among neuroinflammatory, vascular, and neurodegenerative processes in FTD (Zhu et al. 2021; Chu et al. 2025). However, a systematic investigation of the alterations of the ventricular system in a larger FTD cohort and its relationship with peripheral blood biomarkers reflecting these pathophysiological processes is currently lacking.

This study aims to elucidate the role of the ventricular systems in the pathophysiology of FTD. Specifically, we assessed the volumes of the lateral ventricles, inferior lateral ventricles, third ventricle, and fourth ventricle, as well as the asymmetry of the lateral and inferior lateral ventricles and the volume of the choroid plexus. We further sought to determine the correlations between these metrics and clinical scales, brain volumes, and plasma biomarkers. Finally, we explore the mediating factors of the association between the ventricular system and clinical phenotype. We speculate the important roles of the ventricular system in bvFTD and its association with other pathophysiological processes.

## 2 Materials and Methods

### 2.1 Ethics

This cross‐sectional study recruitment took place at the Department of Neurology, Xuanwu Hospital, spanning the period from July 2014 to October 2025. The cohort comprised individuals receiving care as either outpatients or inpatients, alongside healthy volunteers identified via community health screenings conducted during the same timeframe. Before enrollment, written informed consent was secured from every participant. Furthermore, the study protocol was approved by the Institutional Medical Ethics Committee (ethical approval no. 2023200).

### 2.2 Participants

From July 1, 2014, to October 1, 2025, we assembled a cohort of right‐handed individuals drawn from the Department of Neurology at Xuanwu Hospital. The study population consisted of 120 patients diagnosed with behavioral variant frontotemporal dementia (bvFTD) and 87 normal controls (NC). The control group was matched to the patient group based on age, sex, and education. All diagnoses of bvFTD were verified in accordance with the 2011 international diagnostic criteria for probable bvFTD (Rascovsky et al. 2011). Conversely, healthy controls, recruited from community health screenings, demonstrated no signs of cognitive deficits, depression, or anxiety. Their neuropsychological performance fell within standard limits, as evidenced by Mini‐Mental State Examination (MMSE) scores of 24 or higher and a CDR plus NACC FTLD score of 0 in both the behavior/comportment and language domains.

The exclusion criteria were uniformly applied to all subjects: (1) presence of significant neurological or psychiatric conditions capable of influencing cognitive outcomes, such as substance use disorders, alcohol dependence, schizophrenia, intracranial tumors, or cerebrovascular diseases; (2) any standard contraindications to magnetic resonance imaging (MRI); and (3) the unavailability of a reliable informant for the purpose of obtaining clinical history. Additionally, neither group included participants with a history of hypertension, hypercholesterolemia, diabetes, smoking, or previous vascular events. All individuals involved in the study possessed a body mass index (BMI) that fell within the normal range, defined as 18.5–23.9 kg/m^2^.

### 2.3 Clinical Assessments

A comprehensive, standardized battery of neuropsychological tests was administered to all participants. Screening for global cognitive function was performed using the Mini‐Mental State Examination (MMSE) and the Montreal Cognitive Assessment (MoCA), while disease severity was quantified using the CDR plus NACC FTLD scale. To evaluate the severity of behavioral and psychiatric symptoms, the Neuropsychiatric Inventory Questionnaire (NPI‐Q) and Frontal Behavior Inventory (FBI) were utilized; the FBI consists of two distinct subscales: the negative apathy subscale (encompassing items 1–12) and the positive disinhibition subscale (encompassing items 13–24). Finally, functional independence in daily living was measured through the activities of daily living (ADL) scale.

### 2.4 Neuroimaging Data Acquisition

Neuroimaging was obtained at the first assessment. The acquisition of T1‐weighted imaging data was executed using the following parameters: a repetition time (TR) of 6.9 ms, an echo time (TE) of 2.98 ms, and a flip angle of 12°. The inversion time was set to 450 ms, with matrix dimensions of 256 × 256 and a field of view (FOV) covering 256 × 256 mm^2^. The scanning process involved 192 sagittal slices with a thickness of 1 mm and no inter‐slice gap, resulting in a voxel size of 1 × 1 × 1 mm^3^. The total duration for the scan was 4 min and 48 s.

We extracted brain volumes and cortical thickness from MRI scans using FreeSurfer. Preprocessing quality control involved modality alignment and artifact removal. Raw ventricular characterizations—comprising the volumes of the left and right lateral ventricles, left and right inferior lateral ventricles, third and fourth ventricles, and choroid plexus were extracted using FreeSurfer's automated segmentation tool (aseg), followed by visual inspection with particular attention to the anatomical boundaries of the ventricles and choroid plexus. Segmentation errors identified during visual inspection were manually corrected (Figure 1). The total lateral ventricle volume was computed as the sum of the left and right sides. Similarly, the inferior lateral ventricle volume was defined as the sum of the left and right inferior lateral ventricles. To quantify structural asymmetry, we employed a widely used formula (Xu et al. 2024; Sha et al. 2021; Kong et al. 2018):

$$\mathrm{Asymmetry}\mathrm{index}=\left(\mathrm{Left}-\mathrm{Right}\right)/\left(\left[\mathrm{Left}+\mathrm{Right}\right]/2\right)$$

**FIGURE 1:**
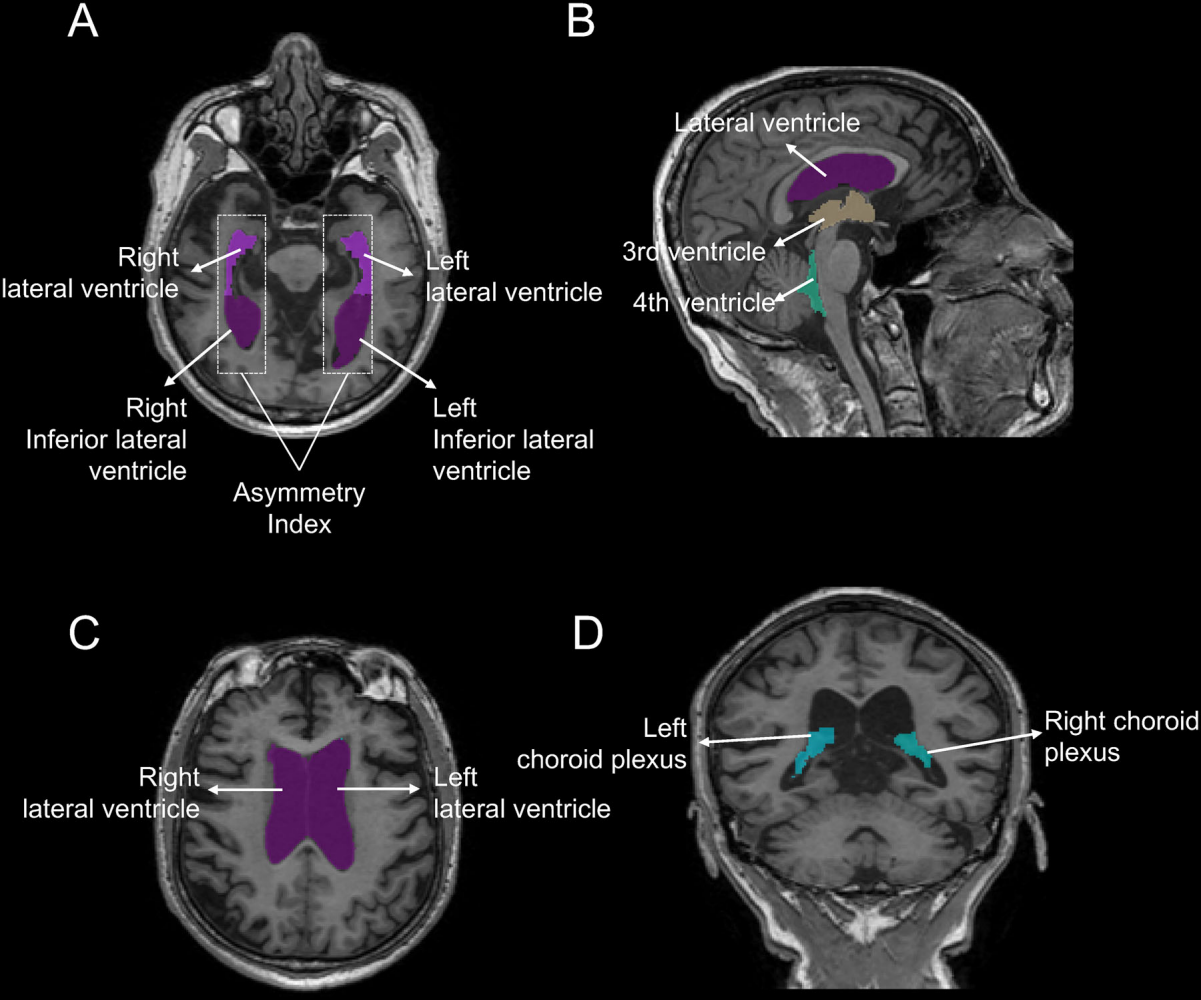
Schematic diagram of the ventricular volume and choroid plexus based on FreeSurfer software. This figure presents a multi‐view anatomical localization and measurement indicators of the cerebral ventricular and choroid plexus volume, providing a standardized anatomical reference for analysis in this study. (A) Global anatomical structure of the ventricular system labels the spatial distribution of the left lateral ventricle, right lateral ventricle, left inferior lateral ventricle, and right inferior lateral ventricle, and asymmetry index; (B) identifies the lateral ventricles, the third and fourth ventricles; (C) hemispheric differentiation of the lateral ventricles; (D) marks the left choroid plexus and right choroid plexus.

According to this definition, a positive asymmetry index reflects a larger volume in the left hemisphere compared to the right.

### 2.5 Plasma Biomarker Analysis

Fasting peripheral blood was collected from 50 FTD patients using EDTA tubes (BD, USA), with all collection and processing steps performed at room temperature at the first assessment (Table S1). Plasma was isolated by centrifugation at 3000 rpm for 10 min, aliquoted, and stored at −80°C until analysis. We employed various immunoassay platforms for biomarker quantification. The S‐PLEX electrochemiluminescence immunoassay (K15639S‐X) was utilized to measure GFAP, NFL, and total tau (t‐tau) following the manufacturer's instructions. Transforming growth factor‐beta 1 (TGF‐β1) was measured using an Absin ELISA kit (abs510032). For other vascular and inflammatory markers, we performed multiplex assays using Luminex xMAP technology. Panel 1 (LXSAHM‐03, R&D system, Minnesota, USA) was used to measure MMP‐9, platelet‐derived growth factor‐BB (PDGF‐BB), galectin‐3, CHI3L1, fractalkine (CX3CL1), VEGFA, and chitotriosidase‐1 (CHIT1). Panel 2 (LXSAHM‐01, R&D system, Minnesota, USA) was used to quantify VCAM‐1. All Luminex assays were conducted according to the manufacturer's protocols at LabEx of UNIV (Shanghai, China), involving incubation with antibody‐coupled magnetic beads, followed by biotinylated detection antibodies and streptavidin‐phycoerythrin. Beads were analyzed on a Luminex X‐200, and sample concentrations were determined using standard curves generated by Milliplex Analyst version 5.1. All assays included quality controls and were performed in batches to minimize inter‐assay variability.

### 2.6 Statistical Analysis

All analyses were conducted using Python 3.11.7 and R 4.0.5, with a two‐tailed *p*‐value of less than 0.05 considered statistically significant. Group comparisons for categorical variables, such as sex, were performed using *χ* ^2^ tests. For continuous variables, independent *t*‐tests or Mann–Whitney *U* tests were applied based on distribution characteristics. Partial correlation analyses, controlling for age, sex, and education, were carried out with false discovery rate (FDR) correction for multiple comparisons. Mediation effects were examined using the mediation package in R, estimating the significance of indirect effects through bootstrap resampling with 5000 iterations. Additionally, receiver operating characteristic (ROC) curves were generated to assess the diagnostic efficacy for FTD, with the area under the curve (AUC), sensitivity, and specificity values calculated.

## 3 Results

### 3.1 Demographics and Clinical Characteristics

A total of 120 bvFTD patients and 87 NC were enrolled in this study. The demographic characteristics of the two groups are summarized in Table 1. There were no significant differences in age (bvFTD: 62.03 ± 6.23 years; NC: 61.03 ± 6.52 years; *p* = 0.2657) or years of education (bvFTD: 8.89 ± 4.25 years; NC: 9.57 ± 2.14 years; *p* = 0.1704) between the bvFTD group and the NC group.

**TABLE 1:** Demographic and clinical characteristics of the study participants.

Characteristics	bvFTD	NC	*p*
*N*	120	87	/
Age	62.03 ± 6.23	61.03 ± 6.52	0.2657
Education	8.89 ± 4.25	9.57 ± 2.14	0.1704
Age at onset	59.71 ± 6.17	/	/
Disease Course	2.27 ± 1.53	/	/
Behavior	2.26 ± 0.84	0	< 0.0001
Language	1.03 ± 0.87	0	< 0.0001
NACC FTLD CDR SOB	10.69 ± 4.88	0	< 0.0001
CDRSOB	7.40 ± 4.38	0	< 0.0001
MMSE	13.98 ± 7.34	28.75 ± 1.27	< 0.0001
MoCA	9.13 ± 6.18	26.23 ± 2.13	< 0.0001
ADL	35.51 ± 14.26	20	< 0.0001
NPIQ	25.85 ± 17.71	0	< 0.0001
FBI apathy	18.41 ± 8.50	0	< 0.0001
FBI disinhibition	7.15 ± 5.99	0	< 0.0001
FBI total	25.56 ± 12.93	0	< 0.0001

Abbreviations: ADL, activities of daily living;  Behavior, Behavioral Score (CDR plus NACC FTLD); bvFTD, behavioral variant frontotemporal dementia;  CDRSOB, the sum of the boxes scores of the 6 domains of the CDR;  FBI, frontal behavioral inventory;  Language, Language Score (CDR plus NACC FTLD); MMSE, Mini‐Mental State Examination;  MoCA, Montreal Cognitive Assessment;  NACC FTLD CDR SOB, the sum of the boxes scores of the 6 domains of the CDR plus the National Alzheimer's Coordinating Center Frontotemporal Lobar Degeneration behavior and language domains;  NC, normal control; NPIQ, Neuropsychiatric Inventory Questionnaire;

In terms of clinical and neuropsychological assessments, the bvFTD group exhibited significantly worse performance across all scales compared to the NC group (all *p* < 0.0001). The mean age at onset of bvFTD was 59.71 ± 6.17 years, with an average disease course of 2.27 ± 1.53 years. Behavioral and language symptoms were prominent in the bvFTD group, with mean scores of 2.26 ± 0.84 and 1.03 ± 0.87, respectively. Cognitive function was severely impaired in bvFTD patients, as evidenced by significantly lower MMSE (13.98 ± 7.34 vs. 28.75 ± 1.27, *p* < 0.0001) and MoCA scores (9.13 ± 6.18 vs. 26.23 ± 2.13, *p* < 0.0001). Functional independence was also compromised, with the bvFTD group showing a significantly higher ADL score (35.51 ± 14.26 vs. 20, *p* < 0.0001). Neuropsychiatric symptoms measured by NPI‐Q were 25.85 ± 17.71. Behavioral abnormalities, measured by the FBI, were marked in bvFTD patients, with total FBI scores of 25.56 ± 12.93, driven by both apathy (18.41 ± 8.50) and disinhibition (7.15 ± 5.99) subscales (all *p* < 0.0001).

### 3.2 Ventricular and Choroid Plexus Morphology Changes in bvFTD

Significant differences in ventricular and choroid plexus features were observed between the bvFTD group and the NC group (Figure 2). Compared to NC, bvFTD patients exhibited marked alterations in all ventricular features, except for the asymmetry index of the lateral ventricle. The inferior lateral ventricles displayed the most pronounced enlargement in bvFTD. Specifically, the left inferior lateral ventricle (bvFTD: 1869.04 ± 1232.46 mm^3^; NC: 393.86 ± 266.66 mm^3^; *t* = 10.97, *p* < 0.0001), right inferior lateral ventricle (bvFTD: 1691.70 ± 1389.92 mm^3^; NC: 386.80 ± 224.98 mm^3^; *t* = 8.6693, *p* < 0.0001), and total inferior lateral ventricle (bvFTD: 3560.74 ± 2171.92 mm^3^; NC: 780.66 ± 450.70 mm^3^; *t* = 11.75, *p* < 0.0001) all showed extremely significant differences. The inferior lateral ventricle asymmetry index was significantly higher in bvFTD (0.1276 ± 0.5981) compared to NC (−0.0320 ± 0.4603; *t* = 2.0806, *p* = 0.0387). For the choroid plexus, both left and right volumes were significantly larger in bvFTD patients compared to NC. The left choroid plexus volume was 952.18 ± 277.43 mm^3^ in bvFTD versus 621.70 ± 246.02 mm^3^ in NC (*t* = 8.87, *p* < 0.0001), and the right choroid plexus volume was 933.20 ± 274.65 mm^3^ in bvFTD versus 607.33 ± 202.89 mm^3^ in NC (*t* = 9.37, *p* < 0.0001).

**FIGURE 2:**
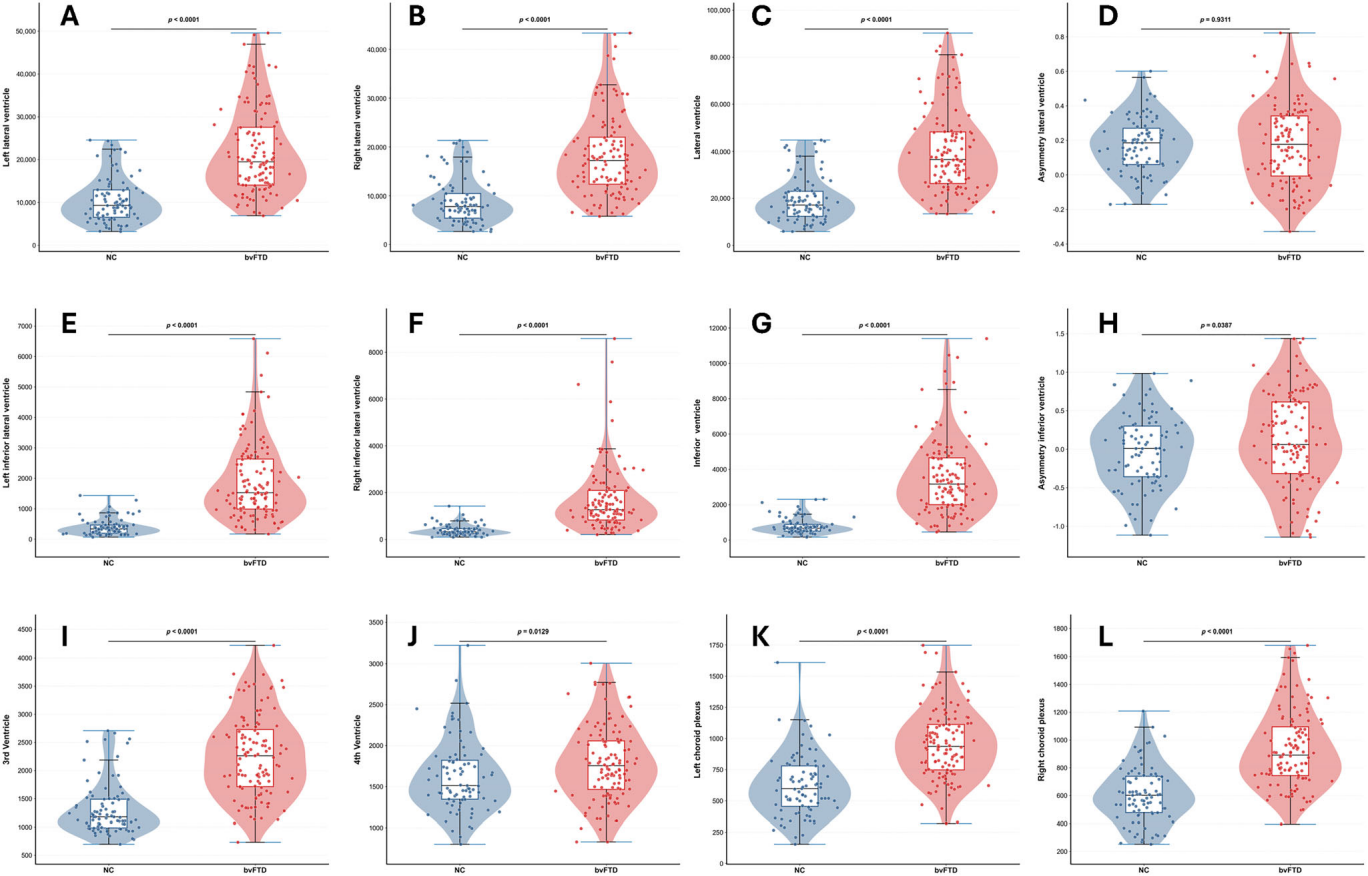
Comparison of ventricular and choroid plexus morphology between the bvFTD and NC groups. All ventricular compartments (A) left lateral ventricle, (B) right lateral ventricle, (C) lateral ventricle, (E) left inferior lateral ventricle, (F) right inferior lateral ventricle, (G) inferior lateral ventricle, (I) third ventricle, (J) fourth ventricle and choroid plexus volumes (K) left choroid plexus and (L) right choroid plexus, exhibited significant enlargement in bvFTD (*p* < 0.0001), with the inferior lateral ventricle showing the most pronounced change. The lateral ventricle asymmetry index (D) showed no significant difference between groups, while the inferior lateral ventricle asymmetry index (H) was significant. bvFTD, behavioral variant frontotemporal dementia; NC, normal control.

### 3.3 Ventricular Features Are Associated With Clinical Scales, Neuroimaging, and Plasma Biomarkers in bvFTD

We found significant associations between ventricular/choroid plexus metrics, brain volumes, clinical scales, and peripheral blood biomarkers (Figure 3, Table 2). With respect to brain volumes, ventricular volumes were negatively correlated with cortical and subcortical gray matter volumes. For instance, left lateral ventricle volume was negatively correlated with total cortical volume (*r* = −0.2922, *p* = 0.0488), while right lateral ventricle volume showed a strong negative correlation with right cortical volume (*r* = −0.4746, *p* = 0.0009). The asymmetry index of the inferior lateral ventricle was positively correlated with right cortical volume (*r* = 0.5710, *p* < 0.0001) and right cerebral white matter volume (*r* = 0.3025, *p* = 0.0410).

**FIGURE 3:**
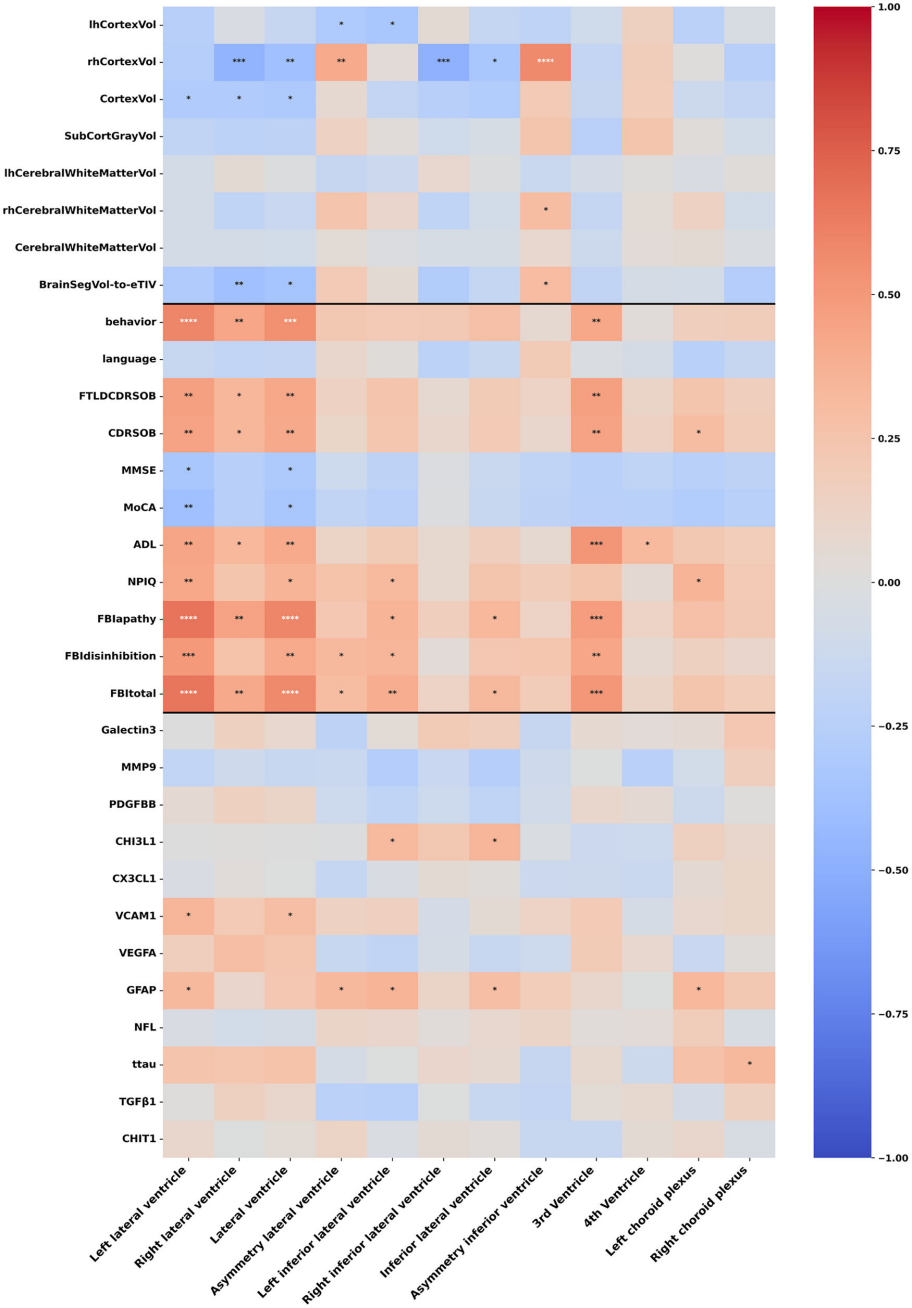
Heatmap of partial correlation analyses between ventricular/choroid plexus metrics, brain volumes, clinical scales, and blood biomarkers. Correlation coefficients (r) are color‐coded, with red indicating positive correlations and blue indicating negative correlations. *p < 0.05, **p < 0.01, ***p < 0.001, and ****p < 0.0001. ADL, activities of daily living; behavior, behavioral score (CDR plus NACC FTLD); BrainSegVol‐to‐eTIV, ratio of brain segmentation volume to estimated total intracranial volume; CDRSOB, clinical dementia rating sum of boxes; CHI3L1, chitinase 3‐like 1; CHIT1, chitotriosidase‐1; CortexVol, total cortical volume; Cerebral White Matter Vol, cerebral white matter volume; CX3CL1, fractalkine; FBI, Frontal Behavior Inventory; FTLD CDR SOB, frontotemporal lobar degeneration clinical dementia rating sum of boxes; Galectin‐3, galectin‐3; GFAP, glial fibrillary acidic protein; language, language score (CDR plus NACC FTLD); lhCortexVol, left hemisphere cortical volume; lh Cerebral White Matter Vol, left cerebral white matter volume; MMP‐9, matrix metalloproteinase‐9; MMSE, mini‐mental state examination; MoCA, Montreal Cognitive Assessment; NFL, neurofilament light; NPIQ, Neuropsychiatric Inventory Questionnaire; PDGF‐BB, platelet‐derived growth factor‐bb; Rh Cerebral White Matter Vol, right cerebral white matter volume; rhCortexVol, right hemisphere cortical volume; SubCortGrayVol, subcortical gray matter volume; TGF‐β1, transforming growth factor‐beta 1; t‐tau, total tau; VCAM1: vascular cell adhesion molecule 1; VEGFA, vascular endothelial growth factor A.

**TABLE 2:** Partial correlation coefficients, raw *p*‐values, and FDR‐corrected *p*‐values between ventricular/choroid plexus metrics and key variables.

X	Y	*R*	*p*‐value	FDR adjusted *p* value
Left lateral ventricle	CortexVol	−0.2922	0.0488	0.2631
Left lateral ventricle	behavior	0.5870	< 0.0001	0.0017
Left lateral ventricle	FTLDCDRSOB	0.4656	0.0011	0.0277
Left lateral ventricle	CDRSOB	0.4488	0.0018	0.0367
Left lateral ventricle	MMSE	−0.3297	0.0252	0.1877
Left lateral ventricle	MoCA	−0.3844	0.0083	0.0967
Left lateral ventricle	ADL	0.4378	0.0023	0.0459
Left lateral ventricle	NPIQ	0.4278	0.0030	0.0483
Left lateral ventricle	FBI apathy	0.6663	< 0.0001	0.0001
Left lateral ventricle	FBI disinhibition	0.4965	0.0004	0.0167
Left lateral ventricle	FBI total	0.6626	< 0.0001	0.0001
Left lateral ventricle	VCAM1	0.3462	0.0184	0.1706
Left lateral ventricle	GFAP	0.3272	0.0264	0.1927
Right lateral ventricle	rhCortexVol	−0.4746	0.0009	0.0246
Right lateral ventricle	CortexVol	−0.2948	0.0467	0.2595
Right lateral ventricle	BrainSegVol‐to‐eTIV	−0.3884	0.0076	0.0917
Right lateral ventricle	behavior	0.4326	0.0027	0.0483
Right lateral ventricle	FTLDCDRSOB	0.3369	0.0220	0.1822
Right lateral ventricle	CDRSOB	0.3389	0.0212	0.1793
Right lateral ventricle	ADL	0.3315	0.0244	0.1855
Right lateral ventricle	FBI apathy	0.4484	0.0018	0.0367
Right lateral ventricle	FBI total	0.4149	0.0041	0.0592
Lateral ventricle	rhCortexVol	−0.3832	0.0086	0.0967
Lateral ventricle	CortexVol	−0.3099	0.0361	0.2239
Lateral ventricle	BrainSegVol‐to‐eTIV	−0.3558	0.0152	0.1533
Lateral ventricle	behavior	0.5418	0.0001	0.0054
Lateral ventricle	FTLDCDRSOB	0.4266	0.0031	0.0483
Lateral ventricle	CDRSOB	0.4183	0.0038	0.0567
Lateral ventricle	MMSE	−0.3089	0.0367	0.2239
Lateral ventricle	MoCA	−0.3403	0.0207	0.1787
Lateral ventricle	ADL	0.4085	0.0048	0.0641
Lateral ventricle	NPIQ	0.3585	0.0144	0.1517
Lateral ventricle	FBI apathy	0.5933	< 0.0001	0.0017
Lateral ventricle	FBI disinhibition	0.4069	0.0050	0.0643
Lateral ventricle	FBI total	0.5743	< 0.0001	0.0021
Lateral ventricle	VCAM1	0.2952	0.0464	0.2595
Asymmetry lateral ventricle	lhCortexVol	−0.3028	0.0408	0.2422
Asymmetry lateral ventricle	rhCortexVol	0.4125	0.0044	0.0605
Asymmetry lateral ventricle	FBI disinhibition	0.3252	0.0275	0.1927
Asymmetry lateral ventricle	FBI total	0.2984	0.0440	0.2533
Asymmetry lateral ventricle	GFAP	0.3258	0.0271	0.1927
Left inferior lateral ventricle	lhCortexVol	−0.3436	0.0194	0.1718
Left inferior lateral ventricle	NPIQ	0.3188	0.0308	0.2011
Left inferior lateral ventricle	FBI apathy	0.3576	0.0147	0.1517
Left inferior lateral ventricle	FBI disinhibition	0.3452	0.0188	0.1706
Left inferior lateral ventricle	FBI total	0.3909	0.0072	0.0896
Left inferior lateral ventricle	CHI3L1	0.3196	0.0304	0.2011
Left inferior lateral ventricle	GFAP	0.3579	0.0146	0.1517
Right inferior lateral ventricle	rhCortexVol	−0.4912	0.0005	0.0178
Inferior lateral ventricle	rhCortexVol	−0.3340	0.0233	0.1846
Inferior lateral ventricle	FBI apathy	0.3357	0.0225	0.1824
Inferior lateral ventricle	FBI total	0.3242	0.0280	0.1927
Inferior lateral ventricle	CHI3L1	0.3477	0.0179	0.1706
Inferior lateral ventricle	GFAP	0.2925	0.0485	0.2631
Asymmetry inferior ventricle	rhCortexVol	0.5710	< 0.0001	0.0021
Asymmetry inferior ventricle	Rh Cerebral White Matter Vol	0.3025	0.0410	0.2422
Asymmetry inferior ventricle	BrainSegVol‐to‐eTIV	0.3094	0.0364	0.2239
3rd Ventricle	behavior	0.4293	0.0029	0.0483
3rd Ventricle	FTLDCDRSOB	0.4654	0.0011	0.0277
3rd Ventricle	CDRSOB	0.4496	0.0017	0.0367
3rd Ventricle	ADL	0.5233	0.0002	0.0088
3rd Ventricle	FBI apathy	0.4791	0.0008	0.0235
3rd Ventricle	FBI disinhibition	0.4305	0.0028	0.0483
3rd Ventricle	FBI total	0.5094	0.0003	0.0124
4th Ventricle	ADL	0.3178	0.0314	0.2011
Left choroid plexus	CDRSOB	0.2980	0.0443	0.2533
Left choroid plexus	NPIQ	0.3535	0.0159	0.1560
Left choroid plexus	GFAP	0.3315	0.0244	0.1855
Right choroid plexus	t‐tau	0.3222	0.0290	0.1961

*Note*: Data are presented as correlation coefficient (*r*) and *p*‐value. All analyses were controlled for age, sex, and education, with FDR correction for multiple comparisons.

Abbreviations: ADL, activities of daily living; behavior, Behavioral Score (CDR plus NACC FTLD); BrainSegVol‐to‐eTIV, ratio of brain segmentation volume to estimated total intracranial volume; CDRSOB, clinical dementia rating Sum of Boxes; CHI3L1, chitinase 3‐Like 1; CortexVol, total cortical volume; FBI, frontal behavior inventory; FDR, false discovery rate; FTLDCDRSOB, frontotemporal lobar degeneration clinical dementia rating sum of boxes; GFAP, glial fibrillary acidic protein; lhCortexVol, left hemisphere cortical volume; MMSE, Mini‐Mental State Examination; MoCA, Montreal Cognitive Assessment; NPIQ, Neuropsychiatric Inventory Questionnaire; Rh Cerebral White Matter Vol, right cerebral white matter volume; rhCortexVol, right hemisphere cortical volume; t‐tau, total tau; VCAM1, Vascular Cell Adhesion Molecule 1.

Regarding clinical symptoms, ventricular volumes were positively correlated with behavioral and cognitive impairment scores. Lateral ventricle volume showed a strong positive correlation with FBI apathy (*r* = 0.5933, *p* < 0.0001) and total FBI score (*r* = 0.5743, *p* < 0.0001), as well as with ADL score (*r* = 0.4085, *p* = 0.0048). The third ventricle volume was strongly correlated with ADL score (*r* = 0.5233, *p* = 0.0002) and FBI total score (*r* = 0.5094, *p* = 0.0003). The asymmetry index of the lateral ventricle was positively correlated with FBI disinhibition (*r* = 0.3252, *p* = 0.0275) and total FBI score (*r* = 0.2984, *p* = 0.0440).

For peripheral blood biomarkers, several ventricular metrics were correlated with plasma inflammatory markers. Left lateral ventricle volume was positively correlated with VCAM1 (*r* = 0.3462, *p* = 0.0184) and GFAP (*r* = 0.3272, *p* = 0.0264). Left inferior lateral ventricle volume showed positive correlations with CHI3L1 (*r* = 0.3196, *p* = 0.0304) and GFAP (*r* = 0.3579, *p* = 0.0146). The right choroid plexus volume was positively correlated with t‐tau (*r* = 0.3222, *p* = 0.0290).

We further investigated the associations between disease duration and ventricular and choroid plexus volumes. Disease duration was positively correlated with left lateral ventricular volume (*r* = 0.307, *p* = 0.038), right lateral ventricular volume (*r* = 0.340, *p* = 0.021), lateral ventricular volume (*r* = 0.341, *p* = 0.020), third ventricular volume (*r* = 0.445, *p* = 0.002), left choroid plexus volume (*r* = 0.309, *p* = 0.037), and right choroid plexus volume (*r* = 0.357, *p* = 0.015). After false discovery rate (FDR) correction, only the correlation between disease duration and third ventricular volume remained statistically significant (Table S2). Additionally, we repeated the correlation analyses between ventricular and choroid plexus morphological measures and clinical characteristics, brain volumes, and plasma biomarkers, additionally adjusting for disease duration alongside age, sex, and education (Table S3).

### 3.4 GFAP Mediates the Association Between the Ventricle and Clinical Features

Mediation analysis was performed to explore whether plasma biomarkers mediated the association between ventricular metrics and clinical symptoms. The results (Table 3) showed that GFAP exerted a significant mediating effect on the relationship between lateral ventricle volume and total FBI score (ACME = 0.0001, *p* = 0.0376), accounting for 13.38% of the total effect. For the left inferior lateral ventricle, GFAP mediated the associations with FBI apathy (ACME = 0.0010, *p* = 0.0190) and total FBI score (ACME = 0.0015, *p* = 0.0228), explaining 31.43% and 27.59% of the total effects, respectively.

**TABLE 3:** Significant results of mediation analysis.

X	Mediator	Y	ACME	ACME *p*	ADE	ADE *p*	Total effect	Total effect *p*	Proportion
Lateral ventricle	GFAP	FBI total	0.0001	0.0376	0.0004	< 0.0001	0.0005	< 0.0001	0.1338
Left Inferior Lateral Ventricle	GFAP	FBI apathy	0.0010	0.0190	0.0021	0.0528	0.0030	0.0036	0.3143
Left Inferior Lateral Ventricle	GFAP	FBI total	0.0015	0.0228	0.0038	0.0200	0.0053	0.0010	0.2759
Inferior lateral ventricle	GFAP	FBI apathy	0.0006	0.0196	0.0013	0.0426	0.0019	0.0032	0.2934
Inferior lateral ventricle	GFAP	FBI total	0.0009	0.0148	0.0018	0.0574	0.0027	0.0036	0.3240

*Note*: Bootstrap resampling (5000 iterations) was used to estimate the significance of indirect effects.

Abbreviations: ACME, average causal mediation effect; ADE, average direct effect; GFAP, glial fibrillary acidic protein.

Additionally, GFAP mediated the relationship between inferior lateral ventricle volume and FBI apathy (ACME = 0.0006, *p* = 0.0196) as well as total FBI score (ACME = 0.0009, *p* = 0.0148), with mediation proportions of 29.34% and 32.40%, respectively.

### 3.5 Ventricular and Choroid Plexus Metrics Show High Diagnostic Values

ROC curve analysis was performed to evaluate the diagnostic value of ventricular and choroid plexus metrics for bvFTD (Figure 4). The inferior lateral ventricle volume exhibited the highest diagnostic efficacy, with an AUC of 0.9550 (95% CI: 0.9274–0.9826, *p* < 0.0001), followed by the left inferior lateral ventricle (AUC = 0.9450, 95% CI: 0.9144–0.9756, *p* < 0.0001) and right inferior lateral ventricle (AUC = 0.9333, 95% CI: 0.8995–0.9671, *p* < 0.0001). The third ventricle also showed good diagnostic performance (AUC = 0.8800, 95% CI: 0.8342–0.9258, *p* < 0.0001), while the lateral ventricle (AUC = 0.8667, 95% CI: 0.8183–0.9150, *p* < 0.0001) and its left/right subdivisions (left: AUC = 0.8617, 95% CI: 0.8125–0.9109, *p* < 0.0001; right: AUC = 0.8560, 95% CI: 0.8058–0.9063, *p* < 0.0001) had moderate to high AUC values. Choroid plexus volumes also demonstrated diagnostic utility, with the right choroid plexus (AUC = 0.8383, 95% CI: 0.7851–0.8915, *p* < 0.0001) outperforming the left choroid plexus (AUC = 0.8248, 95% CI: 0.7694–0.8801, *p* < 0.0001). In contrast, the fourth ventricle showed weak diagnostic efficacy (AUC = 0.6101, 95% CI: 0.5335–0.6866, *p* = 0.0125), while ventricular asymmetry indices exhibited poor diagnostic value: inferior lateral ventricle asymmetry (AUC = 0.5820, 95% CI: 0.5041–0.6599, *p* = 0.0313) and lateral ventricle asymmetry (AUC = 0.5033, 95% CI: 0.4234–0.5831, *p* = 0.9274).

**FIGURE 4:**
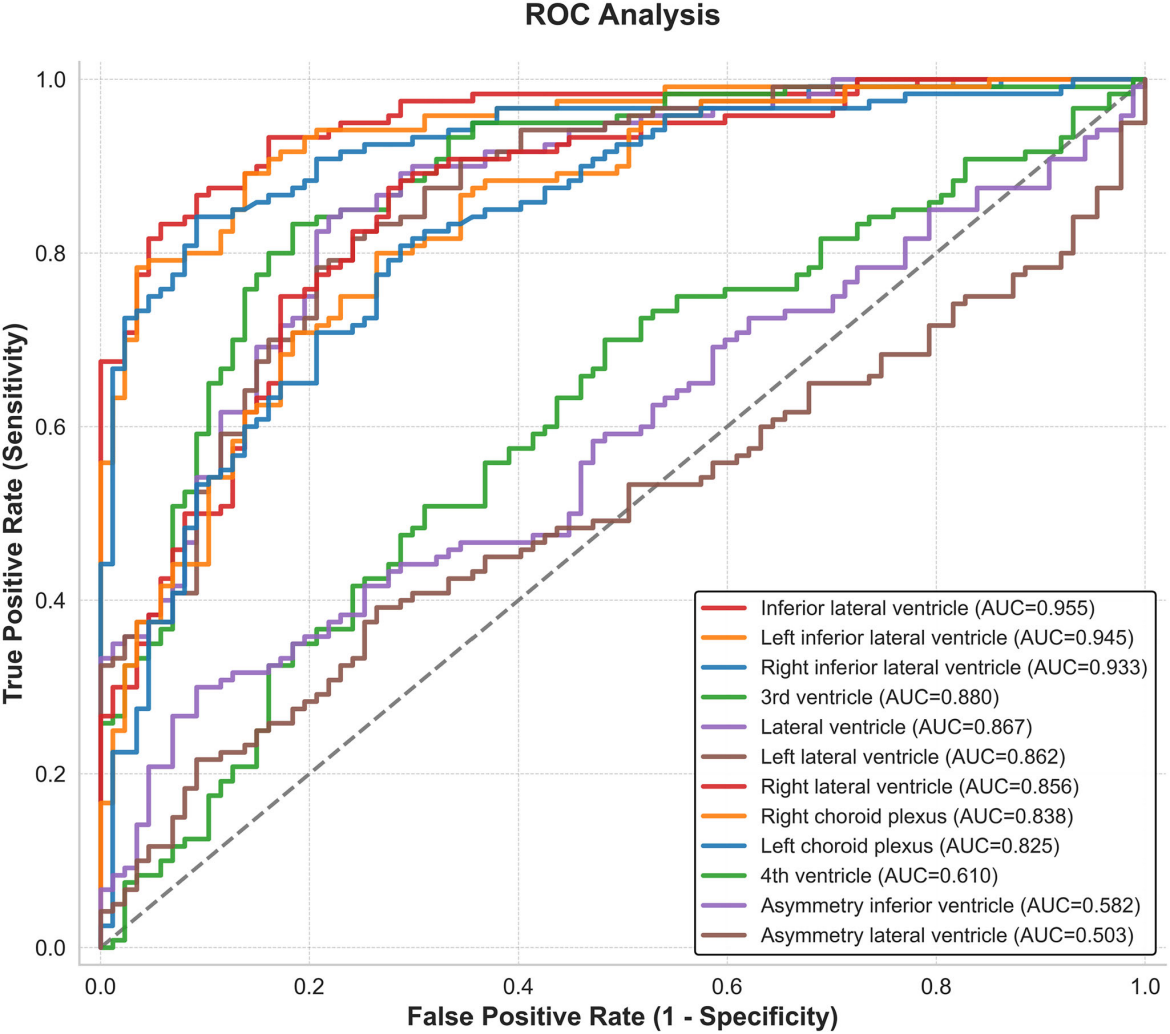
ROC curves of ventricular and choroid plexus metrics for diagnosing bvFTD. As illustrated in the ROC curves, the volume of the inferior lateral ventricle demonstrated the highest diagnostic efficacy (AUC = 0.955, 95% CI: 0.927–0.983), with its left and right subdivisions also performing exceptionally well (left: AUC = 0.945, 95% CI: 0.914–0.976; right: AUC = 0.933, 95% CI: 0.990–0.967). The third ventricle (AUC = 0.880, 95% CI: 0.834–0.926) and the lateral ventricle, along with its hemispheric partitions (total: AUC = 0.867, 95% CI: 0.818–0.915; left: AUC = 0.862, 95% CI: 0.813–0.911; right: AUC = 0.856, 95% CI: 0.806–0.906), also showed good to high diagnostic value, as did the choroid plexus volumes (right: AUC = 0.838, 95% CI: 0.7851–0.8915; left: AUC = 0.825, 95% CI: 0.769–0.880). In contrast, the fourth ventricle showed only weak discriminatory ability (AUC = 0.610, 95% CI: 0.534–0.687), while ventricular asymmetry indices provided no meaningful diagnostic value (inferior lateral ventricle asymmetry: AUC = 0.582, 95% CI: 0.504–0.660; lateral ventricle asymmetry: AUC = 0.503, 95% CI: 0.423–0.583). All AUC values were statistically significant (*p* < 0.05). AUC, area under the curve; ROC, receiver operating characteristic.

## 4 Discussion

This study found that patients with bvFTD exhibit extensive ventricular and choroid plexus alterations, which are correlated with clinical, imaging, and blood biomarkers. Notably, the volume of the inferior lateral ventricle showed the most significant increase and the strongest discriminatory ability between bvFTD patients and healthy controls. Concurrently, mediation analysis identified a statistic mediation effect of GFAP on the association between ventricular enlargement and behavioral abnormalities. These findings suggest that ventricular system morphology may serve as a potential imaging biomarker reflecting disease burden in FTD, and highlight a possible link between astrocyte activation, ventricular enlargement, and behavioral impairment, providing further insights into the pathophysiology of FTD.

### 4.1 Structural Alterations of the Ventricular System in Patients With FTD

This study found that patients with bvFTD exhibit ventricular enlargement and choroid plexus hypertrophy. Consistent with previous studies, ventricular volume expansion has been well‐documented in patients with sporadic and genetic FTD (Knopman et al. 2009; Mahoney et al. 2012; Whitwell et al. 2015). Tavares et al. (2019) also observed ventricular enlargement in presymptomatic patients with genetic FTD, suggesting that alterations in ventricular morphology are potential markers of the early pathological progression of FTD. The significant enlargement of the inferior lateral ventricle observed in this study may be related to its anatomical location—the inferior lateral ventricle is adjacent to the medial temporal structures, and temporal lobe atrophy is one of the early pathological features of bvFTD (Ge et al. 2024).

As the core structure for cerebrospinal fluid (CSF) production and glymphatic system function, the choroid plexus has garnered increasing attention in recent years regarding its association with neurodegenerative diseases. This study discovered an increase in choroid plexus volume in a bvFTD cohort of over 100 individuals, adding new evidence for glymphatic system dysfunction in FTD (Jiang et al. 2023; Xiao et al. 2024; Premi et al. 2024). Xia et al. (2025) found in AD that choroid plexus enlargement is associated with white matter microstructural damage and systemic inflammation. In this study, we observed a similar phenomenon of choroid plexus hypertrophy in bvFTD, which correlated with clinical severity and blood biomarkers GFAP and t‐tau. The potential mechanism may be that choroid plexus hypertrophy leads to abnormalities in CSF circulation and impaired glymphatic clearance, thereby promoting the accumulation of pathological proteins and accelerating the neurodegenerative process (Nedergaard and Goldman 2020; Ringstad et al. 2017).

Additionally, previous studies have demonstrated that CSF dynamics are closely coupled with slow‐wave activity during non‐rapid eye movement sleep, while sleep and circadian rhythm disturbances have also been reported in patients with FTD (Filardi et al. 2024; Fultz et al. 2019; Eschenko et al. 2012). These findings raise the possibility that sleep disturbances may be associated with ventricular and choroid plexus morphological alterations in FTD. Furthermore, growing evidence suggests that the cerebellum is involved not only in cognitive, and behavioral impairment associated with AD and FTD but also in sleep regulation (Canto et al. 2017; Antonioni 2026; Antonioni, Spampinato, et al. 2025). Its potential role in these processes warrants further investigation.

### 4.2 Correlation Between Ventricular Characteristics and Clinical Symptoms

We found that ventricular volumes were positively correlated with the FBI apathy subscale scores, total scores, and ADL scores, while showing a negative correlation with cognitive scale scores such as MMSE and MoCA. This indicates that the degree of ventricular enlargement is closely associated with behavioral abnormalities and functional decline in patients with bvFTD. This result is consistent with the fact that ventricular size is correlated with the severity of dementia (Sapkota et al. 2018).

Notably, the volume of the third ventricle showed the strongest correlation with ADL scores. This may be related to the distribution of limbic system structures surrounding the third ventricle (such as the thalamus and parahippocampal gyrus); atrophy in these regions leads to ventricular enlargement while simultaneously directly impacting patients’ ADL and emotional regulation functions (Mioshi et al. 2013; McKenna et al. 2023). Furthermore, third ventricular enlargement is associated with disease duration, suggesting that it may reflect cumulative neurodegenerative changes over the disease course rather than serving solely as an early marker of tissue loss. This is consistent with previous longitudinal findings reporting that ventricular expansion may accelerate over time (Nestor et al. 2008; Whitwell et al. 2008).

Although the lateral ventricle asymmetry index showed no difference compared to normal controls, within the patient group, it was found to be correlated with the FBI disinhibition subscale. This finding suggested that the imbalance in bilateral ventricular morphology, along with its characteristic features, contributes to behavioral symptoms in bvFTD.

### 4.3 Negative Correlation Between Ventricular Characteristics and Brain Volume

This study confirmed that ventricular volume is significantly negatively correlated with cortical and subcortical gray matter volume, with the right lateral ventricle showing the strongest correlation with right cortical volume. The core pathology of FTD is the progressive loss of neurons in the frontal and temporal lobes, resulting in a reduction of brain parenchyma volume and subsequently leading to passive ventricular expansion. This provides further evidence that the cerebral ventricles are not merely cavities containing protective fluid buffers and waste sinks; their morphology is closely related to central nervous system pathology (Lui et al. 2011; Fame and Lehtinen 2020).

This study found that the inferior lateral ventricle asymmetry index was positively correlated with right cortical volume and right white matter volume, potentially reflecting the characteristic asymmetric neurodegeneration in bvFTD. Previous studies have demonstrated an association between ventricular structural asymmetry and hemispheric metabolic asymmetry in FTD, suggesting that the hemisphere with greater ventricular enlargement may exhibit more pronounced neurodegenerative changes (Jeong et al. 2005). These findings suggest that ventricular asymmetry in bvFTD may primarily reflect underlying asymmetric neurodegeneration rather than representing an independent imaging marker.

### 4.4 Association Between Ventricular System Characteristics and Blood Biomarkers

Plasma biomarkers have emerged as valuable tools: GFAP, CHI3L1, CX3CL, and CHIT1 reflect inflammatory processes in FTD (Xu et al. 2024; Jack et al. 2024). Markers like MMP‐9, VEGFA, VCAM1, PDGF‐BB, TGFβ1, and galectin‐3 indicate various aspects of vascular dysfunction, including remodeling, endothelial activation, and blood‐brain barrier disruption (Chen et al. 2013; Balčiūnas et al. 2009; Cembran et al. 2025; Davis et al. 2025; Souchak et al. 2024). This study is the first to discover that ventricular metrics are associated with various inflammatory and neurodegenerative biomarkers; for instance, left lateral ventricle volume was positively correlated with VCAM1 and GFAP, left inferior lateral ventricle volume was positively correlated with CHI3L1 and GFAP, and right choroid plexus volume was positively correlated with t‐tau.

Although GFAP, a well‐established marker of astrocyte activation, is not specific to FTD and is also elevated in other neurodegenerative disorders, with higher levels in AD than FTD (Chouliaras et al. 2022), increased GFAP levels have been observed in presymptomatic FTD mutation carriers who later developed clinical symptoms and have been associated with cognitive impairment, disease severity, and brain atrophy (Heller et al. 2020, 2021; Sheth et al. 2025). In our study, mediation analysis revealed a significant mediating effect of GFAP on the association between ventricular enlargement and behavioral symptoms, suggesting the critical pathophysiological role of astrocyte activation in FTD. This finding is consistent with previous studies indicating that abnormalities in CSF dynamics are related to inflammatory pathways (Xia et al. 2025). Furthermore, the correlation between CHI3L1, a marker of microglial activation, and inferior lateral ventricle volume further supports the central role of inflammation in the pathology of bvFTD. Similarly, the correlation between t‐tau and choroid plexus volume suggests that choroid plexus enlargement is associated with the severity of neuronal injury.

These findings also highlight the potential complementary value of ventricular morphology, choroid plexus volume, and fluid biomarkers in longitudinal disease monitoring. Previous studies have demonstrated that patients with FTD exhibit greater rates of brain atrophy and ventricular expansion than healthy controls, while alterations in choroid plexus volume have been proposed as a potential early imaging biomarker within the FTLD spectrum (Assogna et al. 2023; A. Antonioni, Granieri, et al. 2025). Changes in fluid biomarkers, such as GFAP and NFL, may occur during the early or even presymptomatic stages of FTD (A. Antonioni, Granieri, et al. 2025; van der Ende et al. 2022). Given the observed associations between plasma biomarkers and ventricular and choroid plexus volumes, future longitudinal studies combining repeated MRI assessments with serial fluid biomarker measurements may help characterize disease trajectories and monitor early neurodegenerative changes, particularly in presymptomatic carriers of pathogenic FTD‐related mutations.

### 4.5 Diagnostic Value of Ventricular and Choroid Plexus Metrics

We further found that the volume of the inferior lateral ventricle is the best indicator for diagnosing bvFTD (AUC = 0.9550). As an extension of the lateral ventricle, the inferior lateral ventricle is adjacent to the medial temporal structures, and temporal lobe atrophy is one of the early pathological features of bvFTD; therefore, its volume changes may more sensitively reflect early pathological injury. Studies based on the GENFI cohort have shown that volumetric differences in the ventricles can be observed as early as 4 years before symptom onset in asymptomatic mutation carriers, and the annual rate of ventricular expansion is higher in asymptomatic carriers than in non‐carriers, suggesting its value as an early diagnostic marker (Tavares et al. 2019).

As we know, ventricular metrics offer several advantages, including reduced image distortion from gradient nonlinearities due to the ventricles’ proximity to the magnet's isocenter and high contrast between ventricles and tissue, which facilitates automated segmentation and implementation in large clinical trials (Tavares et al. 2019). Furthermore, choroid plexus volume demonstrated good diagnostic value, suggesting that it may be associated with FTD progression. In contrast, the diagnostic efficacy of the ventricular asymmetry index was poor, indicating that its value as a diagnostic marker is limited.

This study has certain limitations. First, the sample was derived from a single center, which may introduce selection bias and limit the generalizability of the findings. Therefore, external validation in independent, multicenter, and ethnically diverse cohorts with larger sample sizes is warranted. Second, the cross‐sectional design precludes clarification of the temporal dynamics of ventricular morphology and choroid plexus volume and their association with disease progression. Moreover, the observed mediation effect of GFAP reflects statistical mediation rather than biological causation. Longitudinal studies are therefore needed to clarify these temporal relationships and further validate the observed associations. Third, the relatively small biomarker subgroup may limit statistical power and the robustness of the findings, while the lack of neuropathological confirmation precludes comparisons across distinct pathological subtypes of FTD. Furthermore, given the distinct patterns of brain atrophy reported in genetic FTD (Greaves and Rohrer 2019), the potential influence of genetic heterogeneity on ventricular morphology and choroid plexus volume remains unclear. Future studies involving larger cohorts with well‐characterized genetic and neuropathological profiles are needed to address these limitations. Finally, other dementia subtypes, such as AD and dementia with Lewy bodies, were not included for comparative analysis, making it difficult to ascertain the specificity of these findings of this study.

## 5 Conclusion

This study confirms that patients with bvFTD exhibit significant ventricular enlargement and choroid plexus hypertrophy. The volume of the inferior lateral ventricle serves as a highly sensitive indicator for the diagnosis of bvFTD from healthy controls, and inflammation plays a pivotal role in the association between ventricular morphological changes and behavioral abnormalities. These findings provide potential biomarkers for clinical diagnosis and identify new targets for therapeutic intervention.

## Author Contributions

**Ailing Yue**: conceptualization, investigation, writing – review and editing, writing – original draft, methodology, formal analysis. **Qianqian He**: investigation, validation, writing – review and editing, software, visualization, data curation, writing – original draft. **Hua Lu**: investigation, validation, writing – review and editing. **Yufei Chen**: investigation, validation, writing – review and editing. **Jiahui Hou**: writing – review and editing, validation, investigation. **Yuxuan Xu**: validation, investigation, writing – review and editing. **Liyong Wu**: funding acquisition, writing – review and editing, resources, validation, conceptualization, supervision. **Min Chu**: writing – review and editing, resources, funding acquisition, validation, conceptualization, supervision, project administration.

## Funding

This work was supported by the Beijing Natural Science Foundation (grant no.: 7242067) and the National Natural Science Foundation of China (grant no.: 82401665)

## Conflicts of Interest

The authors declare no conflicts of interest.

## Supporting information




**Supporting Information**: brb371892‐sup‐0001‐SuppMat.docx

## Data Availability

The data that support the findings of this study are available from the corresponding author upon reasonable request.
