# Supplementary material for: Ventricular and Choroid Plexus Morphological Alterations and Their Associations With Peripheral Biomarkers in Behavioral Variant Frontotemporal Dementia
Source: Brain Behav. 2026 Nov 5;16(11):e71892. doi: 10.1002/brb3.71892 (PMC13740348; doi:10.1002/brb3.71892)
Supplement: Supplementary file 1 [file BRB3-16-e71892-s001.docx]

**Table S1 Availability of MRI and plasma biomarker data in the study cohort.**

| Data availability | bvFTD | HC | Total |
| --- | --- | --- | --- |
| Participants enrolled, n | 120 | 87 | 207 |
| Participants with available MRI data, n | 120 | 87 | 207 |
| Participants with available plasma biomarker, n | 50 | **\** | 50 |
| Participants with both MRI and plasma biomarker, n | 50 | **\** | 50 |

**Abbreviations:** bvFTD, behavioral variant frontotemporal dementia; HC, healthy controls; MRI, magnetic resonance imaging;

**Table S2 Partial correlation coefficients, raw p-values, and FDR-corrected p-values between ventricular/choroid plexus metrics and disease duration.**

| ventricular/choroid plexus metrics | R | p value | FDR adjusted p value |
| --- | --- | --- | --- |
| Left lateral ventricle | 0.3066 | **0.0382** | 0.0764 |
| Right lateral ventricle | 0.3404 | **0.0206** | 0.0619 |
| lateral ventricle | 0.3410 | **0.0204** | 0.0619 |
| Asymmetry lateral ventricle | -0.1240 | 0.4117 | 0.4954 |
| Left inferior lateral ventricle | 0.1031 | 0.4954 | 0.4954 |
| Right inferior lateral ventricle | 0.1456 | 0.3342 | 0.4954 |
| Inferior lateral ventricle | 0.1662 | 0.2697 | 0.4623 |
| Asymmetry inferior ventricle | -0.1162 | 0.4418 | 0.4954 |
| 3rd_Ventricle | 0.4450 | **0.0019** | **0.0233** |
| 4th_Ventricle | 0.1078 | 0.4758 | 0.4954 |
| Left choroid plexus | 0.3090 | **0.0367** | 0.0764 |
| Right choroid plexus | 0.3570 | **0.0149** | 0.0619 |

Data are presented as correlation coefficient (r) and p-value. All analyses were controlled for age, sex, and education, with FDR correction for multiple comparisons.

**Table S3 Partial correlation coefficients, raw p-values, and FDR-corrected p-values between ventricular/choroid plexus metrics and key variables.**

| X | Y | R | p value | FDR adjusted p value |
| --- | --- | --- | --- | --- |
| Left lateral ventricle | BrainSegVol_to_eTIV | -0.2974 | 0.0473 | 0.3147 |
| Left lateral ventricle | behavior | 0.5715 | 0.0000 | 0.0041 |
| Left lateral ventricle | FTLDCDRSOB | 0.3682 | 0.0128 | 0.2169 |
| Left lateral ventricle | CDRSOB | 0.3448 | 0.0204 | 0.2367 |
| Left lateral ventricle | ADL | 0.3342 | 0.0249 | 0.2642 |
| Left lateral ventricle | NPIQ | 0.3771 | 0.0107 | 0.2088 |
| Left lateral ventricle | FBIapathy | 0.6220 | 0.0000 | 0.0011 |
| Left lateral ventricle | FBIdisinhibition | 0.4533 | 0.0018 | 0.0657 |
| Left lateral ventricle | FBItotal | 0.6190 | 0.0000 | 0.0011 |
| Left lateral ventricle | VCAM1 | 0.3486 | 0.0189 | 0.2270 |
| Left lateral ventricle | GFAP | 0.3014 | 0.0442 | 0.3147 |
| Right lateral ventricle | rhCortexVol | -0.4876 | 0.0007 | 0.0281 |
| Right lateral ventricle | BrainSegVol_to_eTIV | -0.4058 | 0.0057 | 0.1501 |
| Right lateral ventricle | behavior | 0.4060 | 0.0057 | 0.1501 |
| Right lateral ventricle | FBIapathy | 0.3564 | 0.0163 | 0.2270 |
| Right lateral ventricle | FBItotal | 0.3237 | 0.0301 | 0.2869 |
| Lateral ventricle | rhCortexVol | -0.3903 | 0.0080 | 0.1869 |
| Lateral ventricle | CortexVol | -0.3012 | 0.0444 | 0.3147 |
| Lateral ventricle | BrainSegVol_to_eTIV | -0.3712 | 0.0121 | 0.2138 |
| Lateral ventricle | behavior | 0.5237 | 0.0002 | 0.0138 |
| Lateral ventricle | NPIQ | 0.2951 | 0.0491 | 0.3147 |
| Lateral ventricle | FBIapathy | 0.5266 | 0.0002 | 0.0138 |
| Lateral ventricle | FBIdisinhibition | 0.3505 | 0.0183 | 0.2270 |
| Lateral ventricle | FBItotal | 0.5084 | 0.0004 | 0.0193 |
| Lateral ventricle | VCAM1 | 0.2970 | 0.0476 | 0.3147 |
| Asymmetry lateral ventricle | lhCortexVol | -0.3183 | 0.0331 | 0.3010 |
| Asymmetry lateral ventricle | rhCortexVol | 0.4100 | 0.0052 | 0.1501 |
| Asymmetry lateral ventricle | FTLDCDRSOB | 0.3012 | 0.0443 | 0.3147 |
| Asymmetry lateral ventricle | NPIQ | 0.3048 | 0.0418 | 0.3147 |
| Asymmetry lateral ventricle | FBIapathy | 0.3168 | 0.0340 | 0.3010 |
| Asymmetry lateral ventricle | FBIdisinhibition | 0.3731 | 0.0116 | 0.2138 |
| Asymmetry lateral ventricle | FBItotal | 0.3824 | 0.0095 | 0.1972 |
| Asymmetry lateral ventricle | GFAP | 0.3495 | 0.0186 | 0.2270 |
| Left inferior lateral ventricle | lhCortexVol | -0.3372 | 0.0235 | 0.2574 |
| Left inferior lateral ventricle | NPIQ | 0.3039 | 0.0424 | 0.3147 |
| Left inferior lateral ventricle | FBIapathy | 0.3487 | 0.0189 | 0.2270 |
| Left inferior lateral ventricle | FBIdisinhibition | 0.3315 | 0.0261 | 0.2660 |
| Left inferior lateral ventricle | FBItotal | 0.3835 | 0.0093 | 0.1972 |
| Left inferior lateral ventricle | CHI3L1 | 0.3130 | 0.0363 | 0.3143 |
| Left inferior lateral ventricle | GFAP | 0.3486 | 0.0189 | 0.2270 |
| Right inferior Lateral ventricle | rhCortexVol | -0.4899 | 0.0006 | 0.0281 |
| Inferior Lateral ventricle | rhCortexVol | -0.3308 | 0.0265 | 0.2660 |
| Inferior Lateral ventricle | FBIapathy | 0.2969 | 0.0476 | 0.3147 |
| Inferior Lateral ventricle | CHI3L1 | 0.3383 | 0.0230 | 0.2574 |
| Asymmetry inferior ventricle | rhCortexVol | 0.5698 | 0.0000 | 0.0041 |
| Asymmetry inferior ventricle | rhCerebralWhiteMatterVol | 0.3272 | 0.0282 | 0.2763 |
| Asymmetry inferior ventricle | BrainSegVol_to_eTIV | 0.3092 | 0.0388 | 0.3147 |
| Asymmetry inferior ventricle | MMSE | -0.2985 | 0.0464 | 0.3147 |
| Asymmetry inferior ventricle | MoCA | -0.3068 | 0.0404 | 0.3147 |
| 3rd_Ventricle | SubCortGrayVol | -0.2976 | 0.0471 | 0.3147 |
| 3rd_Ventricle | behavior | 0.4030 | 0.0061 | 0.1501 |
| 3rd_Ventricle | ADL | 0.3604 | 0.0150 | 0.2270 |
| 3rd_Ventricle | FBIapathy | 0.3568 | 0.0161 | 0.2270 |
| 3rd_Ventricle | FBIdisinhibition | 0.3638 | 0.0140 | 0.2270 |
| 3rd_Ventricle | FBItotal | 0.4040 | 0.0059 | 0.1501 |
| 4th_Ventricle | ADL | 0.3169 | 0.0339 | 0.3010 |
| Left choroid plexus | NPIQ | 0.2957 | 0.0486 | 0.3147 |
| Left choroid plexus | GFAP | 0.3058 | 0.0411 | 0.3147 |

Data are presented as correlation coefficient (r) and p-value. All analyses were controlled for age, sex, education and disease duration, with FDR correction for multiple comparisons.

Abbreviations: ADL: Activities of Daily Living; behavior: Behavioral Score (CDR® plus NACC FTLD); BrainSegVol-to-eTIV: Ratio of Brain Segmentation Volume to Estimated Total Intracranial Volume; CDRSOB: Clinical Dementia Rating Sum of Boxes; CHI3L1: Chitinase 3-Like 1; CortexVol: Total Cortical Volume; FBI: Frontal Behavior Inventory; FDR: False Discovery Rate; FTLDCDRSOB: Frontotemporal Lobar Degeneration Clinical Dementia Rating Sum of Boxes; GFAP: Glial Fibrillary Acidic Protein; lhCortexVol: Left Hemisphere Cortical Volume; MMSE: Mini-Mental State Examination; MoCA: Montreal Cognitive Assessment; NPIQ: Neuropsychiatric Inventory Questionnaire; rhCerebralWhiteMatterVol: Right Cerebral White Matter Volume; rhCortexVol: Right Hemisphere Cortical Volume; VCAM1: Vascular Cell Adhesion Molecule 1; SubCortGrayVol: Subcortical gray matter volume.
